# Building community climate resilience: A guide for primary care providers

**DOI:** 10.4102/phcfm.v18i1.5482

**Published:** 2026-06-12

**Authors:** Mandisa A. Ndlovu-Tenego, Robert Mash

**Affiliations:** 1 Division of Family Medicine and Primary Care, Faculty of Medicine and Health Sciences, Stellenbosch University, Tygerberg, South Africa; 2 Faculty of Family Medicine and Public Health, University of Botswana, Gaborone, Botswana

**Keywords:** climate resilience, participatory action research, primary health care, community building, stakeholder engagement, community oriented primary care

## Abstract

Climate hazards pose a threat to health and health services throughout Africa. They directly affect the primary care provider’s clinic operations and patient morbidities like vector-borne and heat-related illnesses. Within a community-orientated primary care approach, primary care providers can assist communities to evaluate their vulnerabilities and capacities and identify the key risks. This article is based on a participatory action reflection (PAR) process (reflection, planning, action, observation of action) conducted by the author in Matsaudi Village, Botswana. A small village community on the edge of the Okavango Delta. Under reflection in the PAR cycle, several steps are described: (1) community and stakeholder engagement, (2) information gathering, (3) identification of vulnerabilities, capacities and risks through workshops on exposure to climate hazards, institutional assets, and the pathways from hazards to health and social effects. The process led to an action plan with both short- and long-term prioritised solutions. This stepwise template from Matsaudi Village enables primary health care providers to sustainably build climate-resilient communities.

## Introduction

Climate change has emerged as one of the greatest global threats to human life in the 21st century. It produces interconnected cascades of impacts that disproportionately affect low- to middle-income countries.^1^ Primary care clinicians witness increased incidence of heat-related illnesses, vector-borne diseases, malnutrition, diarrheal diseases and mental health disorders.^2^ These climate-driven morbidities amplify existing health inequities, creating new burdens for already-stretched health systems.^3^

Within the community-orientated primary care (COPC) and broader primary health care (PHC) frameworks, primary care providers are expected to manage beyond individuals to address population-level determinants of health.^4^ They are uniquely positioned to care for individuals and communities. They understand social and environmental determinants, have a trusted standing within the community, and are integrated into local networks. This enables bridging clinical services and community resilience initiatives.^5,6^

Climate resilience is defined as, ‘The ability of a system or community to anticipate, absorb, adapt to and recover from climate change impacts, while maintaining essential functions and structures’.^7^ Embedding climate resilience activities into routine practice aligns with the COPC cycle of assessing health needs in a participatory approach with the local community, prioritising, planning interventions, implementing actions and evaluating outcomes; thereby transforming reactive care into proactive, system-wide prevention with greater impact.^5^

Given the rapid evolution of climate-health evidence, continuous professional development (CPD) is essential for primary care providers to acquire the competencies required for climate-aware practice as part of COPC. Such CPD can equip physicians with practical tools for stakeholder engagement, hazard mapping, risk assessment and co-creation of locally relevant adaptation strategies. This article presents a step-by-step, participatory action framework derived from fieldwork in Matsaudi Village, Botswana, offering a ready-to-use template that primary care providers can adopt, adapt and integrate into their own practices to build community climate resilience.^8^

## Participatory action and reflection

**FIGURE 1:**
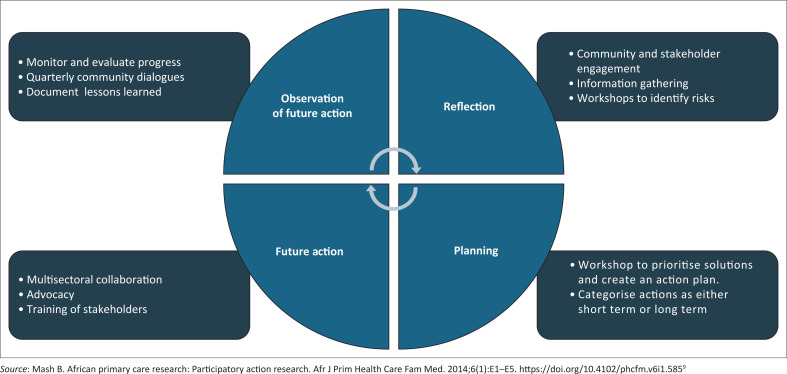
Participatory action research cycle.

The study in Matsaudi Village followed a participatory action research (PAR) cycle, as shown in Figure 1. Participatory action reflection is a tool that has successfully been used to build sustainable climate-resilient communities.^9^ The timeline for the series of workshops was a day long. Each of the validation sessions was half-a-day. The process was spaced over 3 months because of stakeholders’ availability.

## Reflection

### Step 1: Community and stakeholder engagement

This is a vital step as effective community climate resilience work relies and thrives on collaboration. The key is identifying community stakeholders. When engaging the community, learn to understand the local cultural protocols. In many communities, such protocols must be followed by allocating sufficient time for formal greetings, respecting hierarchy, and observance of local customs. During this process, engage stakeholders, understand power dynamics and relationships, and identify what contributions different stakeholders bring to the process. A framework that enables categorisation of who should be recognised, accountable, consulted and informed is called the RACI matrix.^10^

In Matsaudi, meetings with community leadership were our primary means of access. Community leaders included the village chief, headmen, government officials (e.g. the primary school head), and the village development committee. By adhering to protocols, allocating sufficient time to build relationships, and clearly defining roles. Stakeholder engagement enabled us to create social capital for successful PAR research. The researcher was also an important member of the community as she was responsible for primary care in the village and understood their culture and health issues.

After stakeholder identification and categorisation. The team comes together to answer the question, ‘What important problem is affecting us?’ Having a clear, shared vision that links climate resilience actions to health benefits is important. These two key strategies of stakeholder engagement and co-development of a common goal motivate the team to work together towards the common goal of building climate resilience.^11^ Further strategies include the use of the PAR cycle and use of capacity-building activities.

### Step 2: Information gathering

Gathering information builds a comprehensive picture of the community. Below are some data to collect:

- A demographic profile can begin with national census data. Village statistics can be sourced from local leadership structures. Namely, population size, age distribution, household composition and socioeconomic indicators. Such data can identify vulnerable groups within the community.
- Meteorological data can include climate change predictions, local weather patterns and the likelihood of extreme weather events. Collect this from the national meteorological services.
- An inventory of all institutions can be made. These institutions could be assets in building climate resilience.
- Existing data on morbidity, mortality, health facilities and services can be collected from the health information systems or prior research studies. Correlate patterns in the meteorological and health data to better understand the relationship between climate hazards and disease patterns.
- Engage stakeholders to gather qualitative insights into climate events, coping strategies and prevailing hazards.

### Step 3: Identification of vulnerabilities, capacities and risks

A participatory process is used to identify the community vulnerabilities, capacities and risks through a series of workshops. This process was based on the Climate Vulnerability and Capacity Assessment handbook, which has clear guidelines for facilitators.^12^ Facilitating workshops is a skill. Here are some tips:

- Begin each workshop with clear ground rules.
- Consider separate sessions for community members and leaders to minimise power imbalances and encourage open dialogue.
- Make sure that the most vulnerable groups (i.e. women, children, persons living with disabilities) are represented.
- Use visual tools and simple storytelling techniques, particularly important to engage participants with low literacy levels.
- If possible, partner with the non-governmental organisations (NGOs) or academic institutions to provide additional technical support. Keep meetings short and schedule them over several days to respect participants’ time constraints.
- Allow small groups to work respective tasks before reconvening for whole-group synthesis.
- Record decisions on flipcharts so that everyone can validate the outcomes in real time.
- Schedule short debriefs after each session to summarise key points, confirm next steps and gather immediate feedback for iterative improvement.

The following workshops can be conducted.

#### Workshop 1: Hazard-mapping

Participants draw the community, important landmarks and institutional assets. They identify the areas that are exposed to climate hazards such as floods, drought, heat-waves or vector-borne disease, and any hotspots.^12^

The main idea is to help the community identify the areas that are exposed to climate hazards.

**FIGURE 2:**
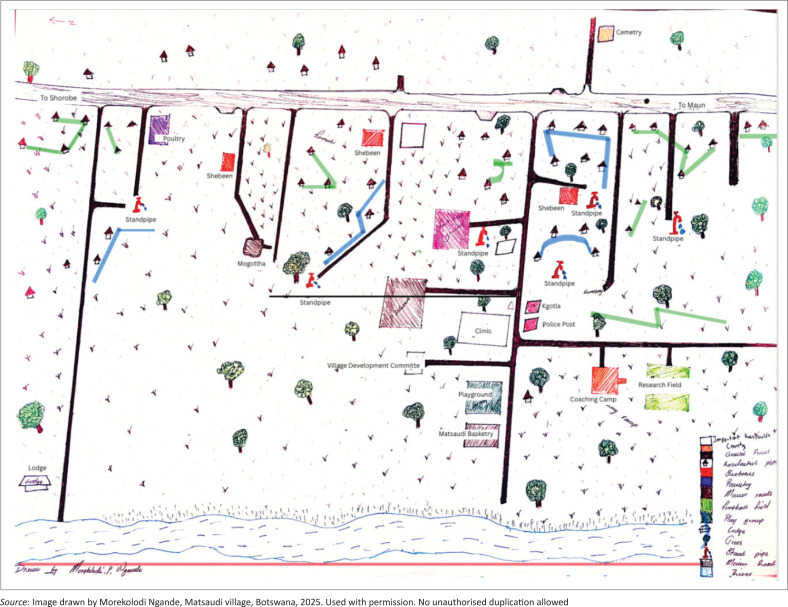
Hazard map showing climate hazards in Matsaudi Village.

Figure 2 is a hazard map that was drawn by the Matsaudi community to identify areas most likely to be affected by hazards. The red coloured marker at the bottom was drawn along the riverbank to indicate the area most affected by floods. The blue coloured marker is located where water tends to be stagnant. And the green coloured marker showed areas most prone to drought, shortages of water and have higher temperatures.

#### Workshop 2: Institutional ecomap

**FIGURE 3:**
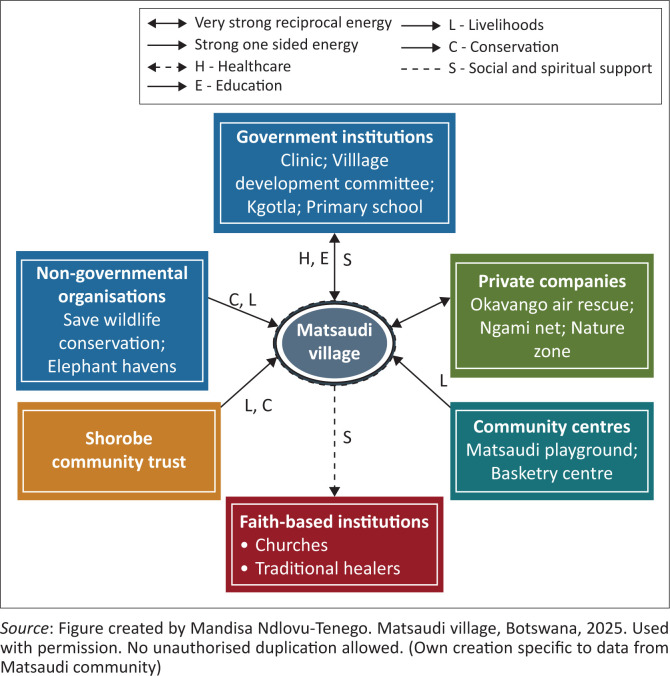
Ecomap diagram of institutional assets in Matsaudi Village.

The ecomap identifies the various institutions that are important in building community climate resilience.^12^ Figure 3 shows institutions in the Matsaudi community and the nature of their relationship with the community. The *most important* institutions were governmental institutions, which gave resources and services to the community, and the community actively supported initiatives from these institutions. The NGOs and community centres were deemed *somewhat important*. The *least important* institutions were the faith-based institutions. The arrows signify how much the institutions contributed to Matsaudi Village and whether partnership was reciprocal or not.

#### Workshop 3: Impact-chain analysis

**FIGURE 4:**
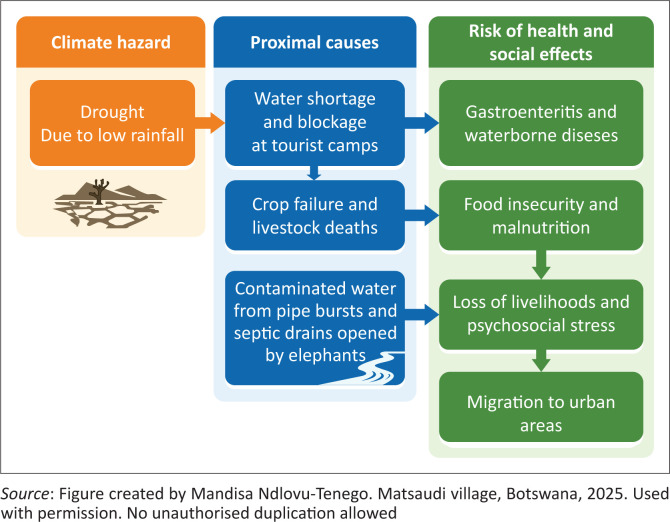
Climate hazard impact chains in Matsaudi community.

In this workshop, participants list hazards by severity and link them to health outcomes.^12^ Figure 4 illustrates how the Matsaudi community linked climate hazards to social and health effects with respect to drought.

### Planning of actions and implementation

After the workshop series, consolidate the findings and present them to the team who were engaged in the workshops for feedback. Use the same visual aids (maps, ecomaps) so participants can see how their inputs shape the final plan. Brainstorm and prioritise potential solutions, thinking of feasibility and the likely impact on climate resilience and health.

Categorise the prioritised solutions into short- and long-term solutions, which are ranked by feasibility and projected impacts on climate resilience, and ultimately health outcomes. Short-term solutions can be led locally (e.g. community education, low-cost gardening), while longer-term solutions may require external support, grant writing or NGO partnerships. For each solution, think of the required resources, specific activities, timeframe and who will be accountable for implementation. Table 1 gives an example from the Matsaudi community.

**TABLE 1:** Example of short- and long-term solutions in Matsaudi community.

Climate hazard	Short-term solution	Who is this assigned to?	Timeline	Long-term solution	Who is this assigned to?	Timeline
Increased temperature and heatwaves	Increase shade in frequently used community spaces like the primary school, the *kgotla* and the clinic	Village development committee, *Kgosi* [Chief] and political leadership of village	6 months to a year	Transition from rain fed farming to solar powered irrigation farming	Village development committee, *Kgosi* [Chief] and political leadership of village	2 years

### Future action

Implement the action plan created in the above step. Advocate for multisectoral collaboration by presenting the plan to district health officers, municipal leaders and development agencies, emphasising the health co-benefits of climate adaptation. Some actions will be immediately implemented with existing resources. While others might require longer terms for planning and additional funding. There are multiple local and global standards that one can further review on the subject, namely Botswana national climate change strategy and World Health Organization guidance on climate-resilient health systems.^13,14,15^

### Observation of future action

Establish a simple monitoring system for the expected activities and outputs over time. Quarterly community dialogues serve as ‘observation points’ where the data are reviewed, successes celebrated and gaps highlighted.^15^ This regular debrief enables rapid refinement of activities and keeps all partners accountable. Documentation of lessons learned, brief case notes, short reports or posts in local health forums creates a living evidence base that can be shared with neighbouring districts or national bodies, thereby amplifying the impact of the project.

Finally, the observation phase should explicitly link back to the start of a new PAR cycle. Prompting a new round of reflection on what worked, what needs adjustment and which emerging climate hazards warrant fresh assessment. By institutionalising these feedback mechanisms, primary care providers transform a one-time project into a sustainable, adaptive process that continuously strengthens community climate resilience.

## Conclusion

This article outlines how a primary care provider could lead a PAR process with their local community with the intention of implementing an action plan to build climate resilience. This process can be conceptualised within a community-orientated model of primary care that focuses on the population at risk and not just individual patients. The cyclical process involves reflection (community engagement, information gathering and risk assessment), planning (prioritising solutions and developing an action plan), taking action (multisectoral action) and observing what happens (monitoring, evaluation and learning).
